# Plasma Gel Matrix as a Promising Carrier of Epigallocatechin Gallate for Regenerative Medicine

**DOI:** 10.3390/jfb15040098

**Published:** 2024-04-10

**Authors:** Takashi Ushiki, Tomoharu Mochizuki, Mami Osawa, Katsuya Suzuki, Tetsuhiro Tsujino, Taisuke Watanabe, Carlos Fernando Mourão, Tomoyuki Kawase

**Affiliations:** 1 Department of Transfusion Medicine, Cell Therapy and Regenerative Medicine, Niigata University Medical and Dental Hospital, Niigata 951-8520, Japan; tushiki@med.niigata-u.ac.jp (T.U.); katsuyasuzu.xq1@nuh.niigata-u.ac.jp (K.S.); 2 Division of Hematology and Oncology, Graduate School of Health Sciences, Niigata University, Niigata 951-9518, Japan; oswmami@clg.niigata-u.ac.jp; 3 Department of Hematology, Endocrinology and Metabolism, Faculty of Medicine, Niigata University, Niigata 951-8510, Japan; 4 Department of Orthopaedic Surgery, Graduate School of Medical and Dental Sciences, Niigata University, Niigata 951-8510, Japan; tomohana@med.niigata-u.ac.jp; 5 Private Practice, Hiroshima 732-0066, Japan; t-tsujino@aaa-plus.jp; 6 Division of Anatomy and Cell Biology of the Hard Tissue, Graduate School of Medical and Dental Sciences, Niigata University, Niigata 951-8514, Japan; watatai@mui.biglobe.ne.jp; 7 Department of Periodontology, Tufts University School of Dental Medicine, Boston, MA 02111, USA; carlos.mourao@tufts.edu; 8 Division of Oral Bioengineering, Graduate School of Medical and Dental Sciences, Niigata University, Niigata 951-8514, Japan

**Keywords:** platelet-poor plasma, plasma gel, epigallocatechin gallate, controlled release

## Abstract

Plasma gel (PG) is a protein matrix prepared from platelet-poor plasma and can be utilized as a drug carrier for controlled release. We previously demonstrated its applicability as a carrier of polyphosphate. Epigallocatechin-3-gallate (EGCG) is the main flavonoid found in green tea and functions as a strong antioxidant. To explore the applicability of PG as an EGCG carrier, we examined the release of EGCG from the PG matrix using an in vitro system. Pooled platelet-poor plasma (PPP) was prepared from four healthy adult male donors, mixed with EGCG, and heated at 75 °C for 10 or 20 min to prepare the PG matrix. The PG–EGCG matrix was incubated in PBS at 37 °C, and the EGCG released into PBS was determined using spectrophotometry. The antioxidant capacity was determined based on the principle of the iodine decolorization reaction. EGCG precipitated and incorporated into the PG matrix during thermal preparation. Trypsin, used to simulate the in vivo degradation of PG, released EGCG from the PG matrix over time. The released EGCG maintained its antioxidant capacity during incubation. These results indicate that thermally prepared PG matrices can be utilized as a promising EGCG carrier in the fields of tissue engineering and regenerative medicine.

## 1. Introduction

Epigallocatechin-3-gallate (EGCG) is the major catechin in green tea and functions as a potent antioxidant, preventing oxidative damage at the cellular level by neutralizing harmful free radicals [1,2,3]. In addition to this effect, extensive studies have demonstrated several pharmacological properties, such as anti-inflammatory, anti-carcinogenic, and neuroprotective effects [4,5]. Thus, to date, EGCG has also been investigated as a potent therapeutic agent. EGCG has been investigated as an adjunct in cancer therapy, as it can induce apoptosis in cancer cells and inhibit tumor angiogenesis [1,2]. Its neuroprotective properties are being explored in the context of neurodegenerative diseases, such as Parkinson’s and Alzheimer’s, where oxidative stress plays a critical role [6]. EGCG also shows promise in topical applications for skin protection and rejuvenation [7].

The half-life of EGCG is ~5 h in the plasma in the case of systemic administration [8]. Furthermore, the chemical stability of EGCG varies with the conditions for the preparation and storage of the solution and the sites for topical application [9]. In brief, EGCG is generally more stable at relatively higher concentrations, under acidic conditions, or at lower temperatures. Thus, innovative applications using carrier materials, combinational treatments, protective agents, and so on are required to augment its efficacy and effectiveness through continuous and efficient delivery to specific sites with minimal systemic adverse effects. To date, several studies have reported montmorillonite [10], liposomes [11], chitosan-β-lactoglobulin complexes [12], and gelatin-γ-polyglutamic acid-based hydrogels [13] as promising carrier materials for EGCG. Each material has both advantages and disadvantages for specific purposes. For example, montmorillonite has a high capacity for the inclusion and retention of EGCG; however, it releases EGCG quickly under gastrointestinal conditions [10]. On the other hand, gelatin-γ-polyglutamic acid-based hydrogels seem superior in their sustained release of EGCG; however, their capacity for EGCG inclusion is relatively low, and the EDC crosslinker has cytotoxic activity at higher concentrations [14]. In addition, these materials require more time to be commercially available in the clinical setting. Thus, carrier materials that meet the requirements specific for topical application in skins, joints, and gingival tissues have not yet been developed.

To develop more biocompatible, high-capacity EGCG carriers, we paid attention to a recently developed and clinically applied biomaterial, plasma gel (PG), that can be easily prepared from autologous blood samples. PG is a protein-based biomaterial prepared from an acellular plasma fraction by means of thermal processing. The advantages of PG include its high biocompatibility, its controllable mechanical stiffness, and its biodegradability. Furthermore, when prepared from autologous blood samples, PG can minimize the risk of possible immune reactions and pathogen transmission, similarly to autologous platelet-rich plasma. Therefore, PG can be used topically as a drug delivery system. Based on this concept, a previous study examined the applicability of PG as a carrier of polyphosphate (polyP) and demonstrated that PG can retain polyP for several days in vitro despite its simple linear configuration [15]. Thus, our research group suggested that PG could more efficiently retain biomolecules with complicated configurations or large sizes for prolonged periods. The PG–EGCG complex can be prepared upon request at the point of care at any time. Using PG as a carrier for EGCG can offer significant advantages over other EGCG delivery systems [1,4,7]. While carriers such as liposomes, nanoparticles, and hydrogels have shown the potential to encapsulate and release EGCG, they often require complex synthesis processes, may cause immune responses, or exhibit limited biocompatibility. Plasma gel, derived from autologous sources, minimizes these concerns by providing a biologically compatible environment that facilitates sustained release [16]. Plasma gel can be easily customized to adjust its mechanical properties and degradation rates, which makes it suitable for targeted delivery and release tailored to specific therapeutic needs. In addition, the mechanical strength of the PG–EGCG complex can be altered depending on the method and purpose of treatment—for instance, fluidic injectable or solid implantable types.

This study aims to improve the effectiveness of EGCG therapy by using a plasma gel (PG) as a carrier for the bioactive molecule [14]. The biodegradable and customizable properties of PG can be used to extend the retention and controlled delivery of EGCG [1,13]. This is particularly useful for localized treatment, as it minimizes systemic exposure and side effects while still exploiting the antioxidant and pharmacological benefits of EGCG. The PG–EGCG complex is a novel approach that aligns with the growing demand for precision in therapeutic interventions and adaptable solutions in the clinical setting. This study sought to confirm that PG can effectively release EGCG in vitro based on previous research on polyphosphate delivery [15]. Thus, considering the advantages of EGCG, the hypothesis of the present study was that the PG matrix could serve as a promising carrier to efficiently deliver EGCG to specific sites. To test this hypothesis, in this initial study, we examined the release of EGCG under in vitro experimental conditions used in a previous study by our research group testing polyP [15].

## 2. Materials and Methods

### 2.1. Blood Sample Collection and Ethical Considerations

This study’s protocol received ethical clearance from the Niigata University Ethics Committee for Human Participants, located in Niigata, Japan. This approval, signified by the project identification code 2019-0423 (approve date: 1 June 2020), was granted in strict adherence to the ethical standards set forth in the Helsinki Declaration, updated in 2013. This declaration provides a global framework for research ethics, emphasizing the protection of human subjects in biomedical research. In compliance with these standards, informed consent was meticulously obtained from every participant, ensuring that they were fully aware of the study’s nature, potential risks, and benefits. This comprehensive ethical review process underscored the commitment to upholding high ethical standards and respect for all individuals who contributed to this study.

Peripheral blood samples were collected from six healthy male volunteers whose ages spanned from 25 to 64 years. Blood was collected using glass vacuum blood collection tubes (Vacutainer^®^; BD Biosciences, Franklin Lakes, NJ, USA) containing a formulation of acid–citrate–dextrose solution (ACD-A). Following collection, the blood underwent a centrifugation process, a technique detailed in a previous study [15], with the precise goal of preparing platelet-poor plasma (PPP). These PPP samples were mixed to created “pooled PPP” and stored at −20 °C until use.

### 2.2. Heating Protocol

EGCG (TCI Chemical Industry Co., Ltd., Tokyo, Japan) was dissolved in Milli-Q water with intermittent gentle agitation in a water bath (50 °C) for a short time (~1 min) and stored in a cool dark place. When EGCG precipitated during storage, the stock solution was agitated quickly in a water bath before addition to PPP.

Pooled PPP samples (100 μL) in 2 mL plastic microtubes were mixed well with 10 μL or 15 μL of 20 mM EGCG and left for 10 min at room temperature (21−23 °C), heated in a heating block at 75 °C for 10 min, and cooled under laminar flow on a clean bench at room temperature for 10 min. This process is important for promoting the evaporation of water from the freshly prepared PG matrix. PPP gels were then subjected to a subsequent EGCG-release test as described previously [15] and below. 

### 2.3. EGCG-Releasing Test

One milliliter of PBS was added to each PG matrix and incubated at 37 °C in a heat block fry bath (TAITEC, Koshigaya, Japan) for up to 8 days. Aliquots (10 μL) of the samples were collected every 24 h for quantitative analysis. To simulate in vitro proteolytic conditions, 2.5% trypsin was added to PBS to obtain a final concentration of 0.025%. 

In parallel, the degradation of the PG matrix was photographed using an Olympus digital CMOS camera (Stylus TG-2 Tough, Olympus, Tokyo, Japan).

### 2.4. Quantitative Analysis of EGCG Using a Spectrophotometer

An aliquot (10 μL) of PBS from the PG–EGCG incubation samples was mixed with 140 μL of 20 mM l-arginine (FUJIFILM Wako Pure Chemicals, Osaka, Japan) by means of vortexing and incubated for 30 min at room temperature [17]. To optimize the wavelength for the quantification, the absorption values were measured over a wide spectrum of wavelengths (250–500 nm) using polystyrene cuvettes and a spectrophotometer (Smartspec Plus; Bio-Rad, Hercules, CA, USA) in the absence and presence of PPP. For the determination of EGCG level in each sample, the absorbance was measured at 325 nm. When the predicted EGCG concentrations exceeded the range of the standard curve, aliquots were appropriately diluted with PBS.

### 2.5. Scanning Electron Microscopic Examination of Plasma Gel

To maintain their microstructure, PG matrices mixed with or without EGCG were initially fixed using a 2.5% neutralized glutaraldehyde solution for 2 h at room temperature and cut into pieces for a further 1 h of fixation with fresh glutaraldehyde solution. The PG matrices were then dehydrated in a series of ethanol solutions, treated with t-butanol, and frozen at −20 °C for 30 min. After freeze-drying, the prepared samples were sputter-coated and examined using a high-resolution scanning electron microscope (SEM) (TM-1000; Hitachi, Tokyo, Japan) operating at an acceleration voltage of 15 kV [15].

### 2.6. Evaluation of the Antioxidant Capacity of the PBS Samples Using a Spectrophotometer

In a preliminary study, degraded PG was found to have significant antioxidant capacity. Therefore, to distinguish the EGCG-dependent decolorization from the PG-dependent decolorization, the content of EGCG (the original: 10 μL in 100 μL PPP) was increased by approximately three times, and the volume of PBS (the original: 1000 μL) was decreased by two times. Thus, pooled PPP samples (70 μL) in 2 mL plastic microtubes were mixed well with 30 μL of 20 mM EGCG (or Milli-Q) and left for 10 min at room temperature (21−23 °C), heated in a heating block at 75 °C for a prolonged period of time, 20 min, and cooled under laminar flow on a clean bench at room temperature for 10 min. The resulting PG matrices were incubated in 500 μL of PBS at 37 °C. To degrade the PG matrix, 0.025% trypsin was added to PBS.

The antioxidant capacities of the samples were evaluated based on the principle that iodine can be converted to iodine ions and decolorized under antioxidant conditions. As described in the quantitative analysis of EGCG released in PBS (Section 2.4), an aliquot (20 μL) of PBS from the PG–EGCG-incubated samples was mixed with 80 μL of 3.125 mg/mL povidone-iodine (available iodine = 0.3125 mg/mL) (Meiji Seika Pharma Co. Ltd., Tokyo, Japan). Before determining the antioxidant capacity, each sample was diluted 3.5 times with Milli-Q water, and the absorbance was measured at 400 nm.

### 2.7. Qualitative Assay of the pH of the PBS Samples Using a pH Indicator Strip

To confirm the effects of the acidic anticoagulant on the pH of the PBS, 5 μL PBS samples were dripped onto the pH indicator strip (MACHEREY-NAGEL GmbH & Co. KG, Dueren, Germany). Immediate color changes in the center indicator paper were photographed using the digital CMOS camera. 

### 2.8. Statistical Analysis

To prepare standard curves, each EGCG concentration was determined in duplicate, and each mean value was plotted on a graph. To compare these regression lines and evaluate the possible effects of PPP on spectrophotometric EGCG determination, one-way ANCOVA was performed using SigmaPlot (version 14.5; Systat Software, Inc., Palo Alto, CA, USA).

Data on EGCG release are expressed as the mean ± SD. To quantify differences in EGCG release over time between groups, one-way ANOVA with post hoc Dunnett’s multiple comparison test was performed. When the normality test or the equal variance test failed, one-way ANOVA based on the rank test was performed. The comparison was conducted between each control (day 1). Differences were considered statistically significant at *p* < 0.05.

## 3. Results

**Figure 1:**
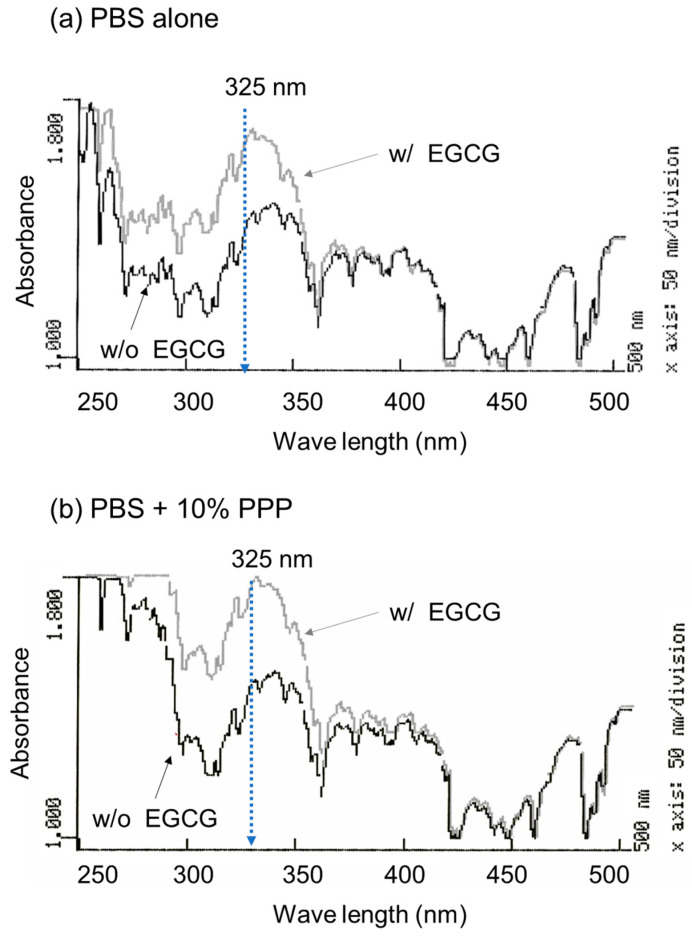
The absorption spectra of PBS containing epigallocatechin gallate (EGCG) in the absence (**a**) and presence of 10% platelet-poor plasma (PPP) (**b**). The absorption of the sample without (black lines) and with 10 μM EGCG (grey lines) was examined in the range of 250 to 500 nm.

To optimize the wavelength for the spectrophotometric determination of EGCG levels, the absorption spectrum was examined in the absence and presence of EGCG, as shown in Figure 1. A comparison of the absorption spectra in the range of 250–500 nm in the absence of PPP is shown in Figure 1a. In the range of approximately 270–350 nm, 10 μM EGCG significantly increased the absorption. Although basal absorption was much higher in the ultraviolet region (<300 nm), a similar observation was obtained in the presence of 10% (*v*/*v*) PPP (Figure 1b).

**Figure 2:**
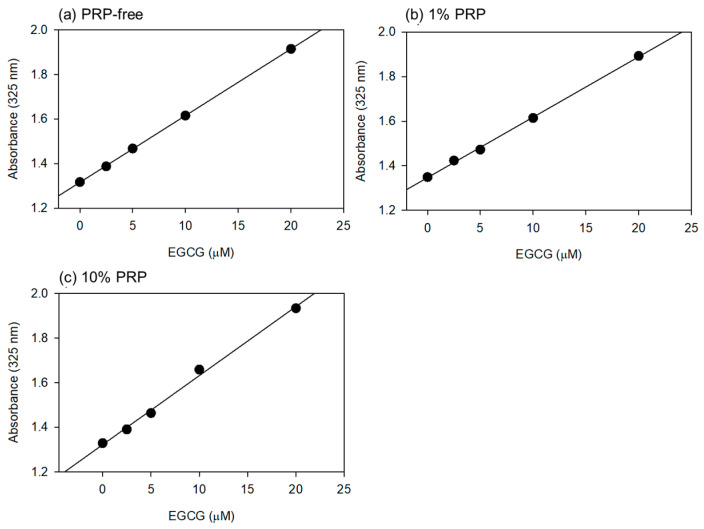
The standard curves for epigallocatechin gallate (EGCG) in PBS containing 1% (**b**) and 10% (*v*/*v*) (**c**) platelet-poor plasma (PPP). In the control (**a**), PPP was not added to PBS. Plasma gel was not used for this quantitative analysis. Each point represents the average of duplicate determination.

Based on the data shown in Figure 1, the optimal wavelength for the quantification of EGCG levels was fixed at 325 nm. Standard curves were drawn in the absence and presence of 1% and 10% PPP (Figure 2). Visually similar lines were found, and the values for the coefficient of determination (R2) were calculated to be 1.000 (a), 0.999 (b), and 0.996 (c). The possible differences were further tested using one-way ANCOVA, and no significant differences were detected among the lines.

**Figure 3:**
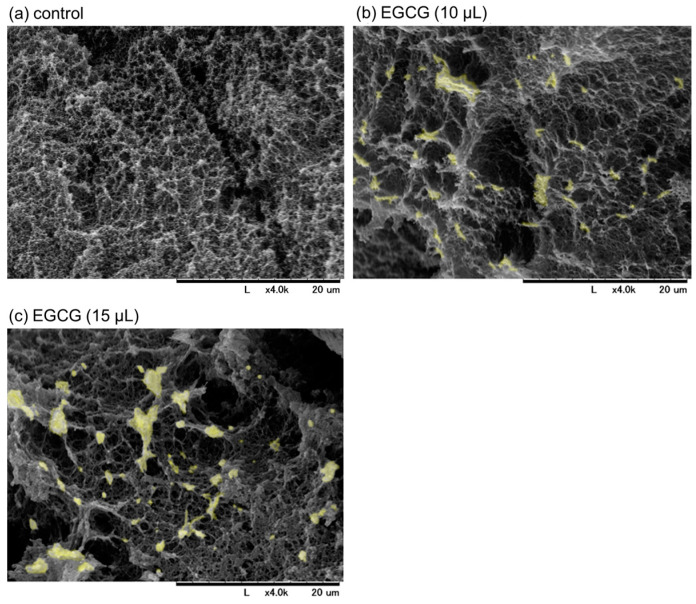
The scanning electron microscopy images of freshly prepared plasma gel (PG) matrices containing approximately 10% (**b**) and 15% (*v*/*v*) (**c**) of 20 mM epigallocatechin gallate (EGCG) in platelet-poor plasma. The PG shown in (**a**) contained no EGCG. The cross-sections of the individual PG matrices were examined. The microstructures, which were not detected in the control, are marked in light yellow in (**b**,**c**). Similar findings were obtained from three independent experiments.

SEM images of freshly prepared and immediately fixed PG matrices with or without EGCG are shown in Figure 3. Examination of the cross-sectional areas revealed that particular microstructures (marked in light yellow), which had not yet been identified but were probably composed of precipitated EGCG, were found in the PG matrices containing EGCG (Figure 3b,c vs. Figure 3a). In the presence of 15 μL (approximately 15%) of 20 mM EGCG, EGCG-containing structures and porosity increased, as indicated by the aperture among fiber-like constructs. As a result, the resulting PG matrix was easily degraded in PBS, even in the absence of trypsin. Thus, a PG matrix containing 10% (*v*/*v*) 20 mM EGCG was examined in subsequent experiments.

**Figure 4:**
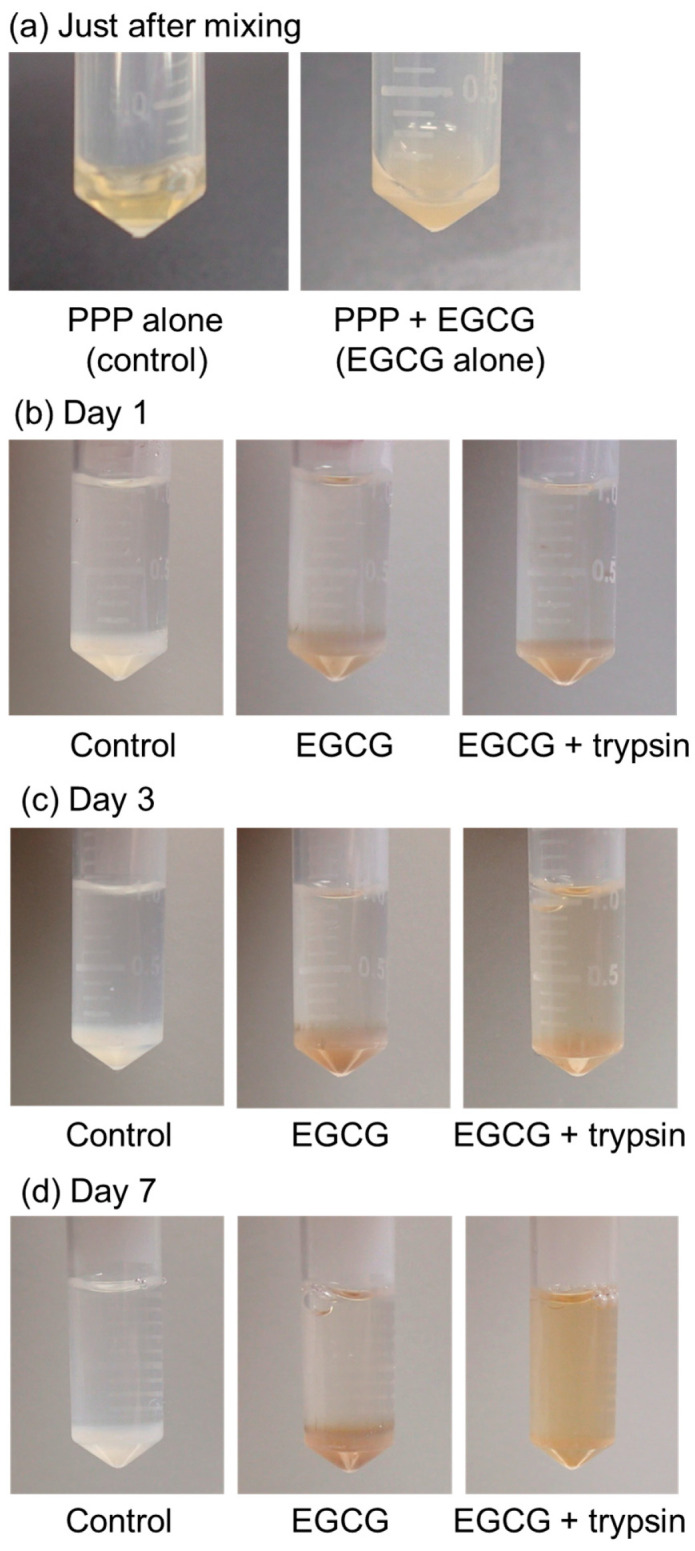
The time-course of changes in plasma gel (PG) matrices containing epigallocatechin gallate (EGCG) in PBS in the presence of 0.025% (*v*/*v*) trypsin at 37 °C. (**a**) Just after mixing, (**b**) day 1, (**c**) day 3, and (**d**) day 7 of incubation. Similar findings were obtained from three independent experiments.

The time-course changes in the appearance of the PG matrices containing EGCG are shown in Figure 4. A stock solution of 20 mM EGCG in Milli-Q water was obtained. However, the mixture became clouded and precipitated EGCG immediately after the addition of the stock EGCG solution to PPP (Figure 4a). The PG matrix containing EGCG exhibited a light brown color compared to that of the control without EGCG (white color) (Figure 4b). In the absence of 0.025% (*v*/*v*) trypsin, no appreciable degradation of PG matrices was observed until 7 days of incubation (Figure 4b–d). In contrast, the degradation of PG matrices occurred after at least 3 days of incubation, and the PG matrices were almost fully degraded within 7 days (Figure 4d).

**Figure 5:**
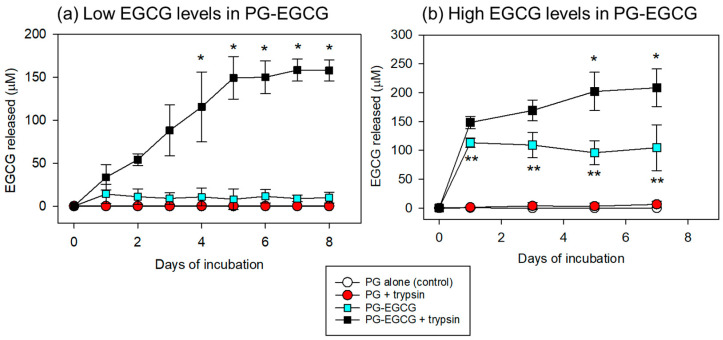
Time-course changes in the levels of EGCG released from the PG matrix into the PBS. (**a**) In the PG matrices containing low EGCG levels, trypsin significantly increased EGCG release on and after day 4. * *p* < 0.05 (n = 6) compared with the control on day 1. (**b**) To evaluate the net EGCG antioxidant capacity (see Figure 6), the preparation protocol for the PG–EGCG matrix was modified as described in the Materials and Methods section. In the PG matrices containing high EGCG levels, trypsin significantly increased EGCG release on and after day 5. * *p* < 0.05 (n = 6) compared with the control on day 1. ** *p* < 0.05 (n = 6) compared with the PG-EGCG with trypsin group at the same time points.

Under these conditions, EGCG release was quantified and compared between the groups. The time-course changes in the EGCG levels in PBS are shown in Figure 5. In the case of low EGCG levels in the PG matrix (10 μL of EGCG added to 100 μL of PPP), significant EGCG release was observed only in the PG–EGCG group with trypsin (Figure 5a). In the absence of trypsin, EGCG release was not observed. In addition, in the presence of 0.025% trypsin, EGCG was not detected in the EGCG-free PG matrix group, either. Trypsin gradually increased EGCG release from the PG–EGCG matrix in a time-dependent manner. Significant differences (vs. the control on day 1) were obtained on and after day 4 (*p* < 0.05). 

In the case of high EGCG levels in the PG matrix (30 μL EGCG added to 70 μL PPP), EGCG was released into the PBS (Figure 5b). As observed in the low EGCG case, EGCG was not detected in the PBS of the EGCG-free samples. In the PG–EGCG samples, EGCG was released immediately, regardless of trypsin; however, trypsin induced the burst of EGCG release in the initial 24h and thereafter gradually increased EGCG release. Significant differences (vs. the control on day 1) were obtained on and after day 5 (*p* < 0.05). For the comparisons between the PG–EGCG without trypsin and the PG–EGCG with trypsin groups, significant differences were observed from day 1 to day 7. 

**Figure 6:**
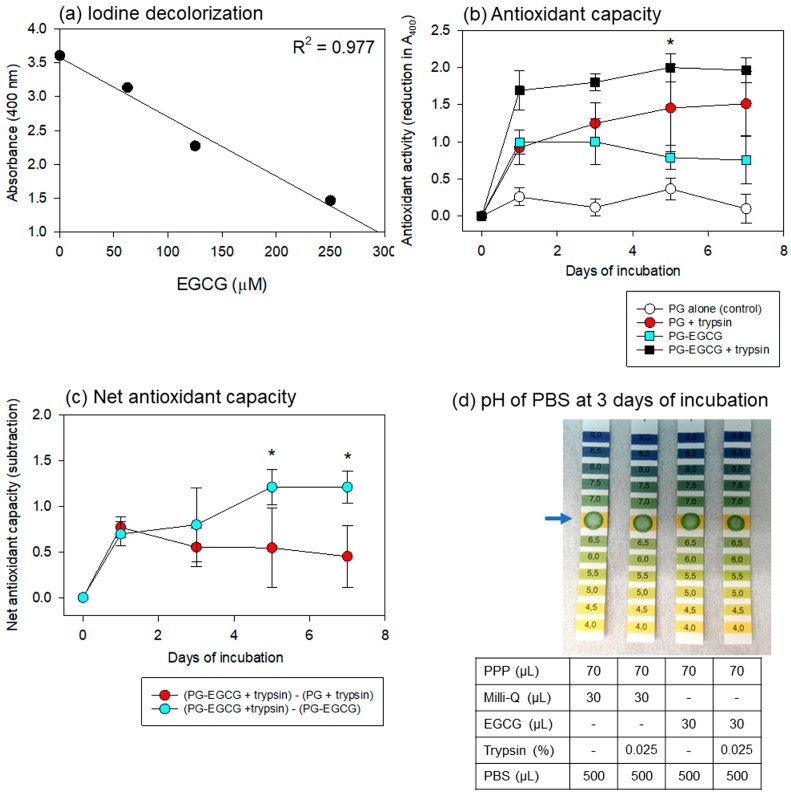
Standard curve of iodine decolorization (**a**), antioxidant capacity of PBS samples (**b**,**c**), and pH of PBS samples (**d**). (**a**) Linearity was obtained in this range of the standard curve using an authentic EGCG reagent. Data are representative of three independent experiments. (**b**) Time-course changes in the antioxidant capacity of the PG matrix alone, PG matrix with trypsin, EGCG-containing PG matrix without trypsin, and EGCG-containing PG matrix with trypsin were recorded in 500 μL PBS at 37 °C. * *p* < 0.05 (n = 6) compared with the corresponding group on day 1. (**c**) Time-course changes in the net antioxidant capacities of EGCG and PG calculated from the data in panel (**b**). * *p* < 0.05 (n = 6) compared with the counterpart at the same time points. (**d**) The effects of the anticoagulant, ACD-A, and other additives on the pH of the PBS samples. The data are representative of the data from repeated experiments.

Regarding the iodine-based spectrophotometric assay for antioxidant capacity, Figure 6 shows the linearity of the standard curve using EGCG, the time-course changes in the antioxidant capacity of the PBS samples, and the pH of the PBS samples. To obtain a wider range of linearity, dilutions of the original povidone-iodine solution and iodine-sample mixtures were optimized. As shown in Figure 6a, a 32-fold dilution of the original iodine solution and a 3.5-fold dilution of the mixture were found to be the most appropriate for our purpose.

For the antioxidant capacity assay, the PG matrix containing high levels of EGCG was examined under the same conditions. Time-course changes in the antioxidant capacity of the PBS samples are shown in Figure 6b. The PG–EGCG with trypsin group exhibited the highest antioxidant capacity. As EGCG was also released non-enzymatically, significant levels of antioxidant capacity were observed in the PG–EGCG without trypsin group. Surprisingly, the EGCG-free PG matrix exhibited antioxidant activity in the presence of trypsin. This capacity increased gradually, but not significantly, with time. No significant antioxidant capacity was observed in the EGCG-free PG matrix without trypsin (control).

Based on these data, the net antioxidant capacities of EGCG and PG were calculated (Figure 6c). The net EGCG antioxidant capacity (represented by red circles) was sustained but did not significantly decrease at similar levels throughout the incubation period. In contrast, the sum of the degraded PG plus released EGCG antioxidant capacity (represented by light blue circles) significantly increased with time, probably due to the degradation of the PG matrix.

The effects of the anticoagulant, ACD-A, and other additives on the pH of the PBS added to the PG matrices are shown in Figure 6d. After 3 days of incubation, the pH of all PBS samples was not acidic, and no substantial differences were observed among the groups. These trends were observed throughout the incubation period.

## 4. Discussion

The present research underscores the plasma gel (PG) matrix’s efficacy as a novel delivery system for EGCG, offering a nuanced understanding of its controlled release capabilities. The pivotal discovery that EGCG release is notably enhanced in proteolytic conditions, particularly with trypsin’s inclusion, marks a significant step forward in drug delivery research. To help readers’ understanding, it is noted that trypsin is not included to release EGCG in PG–EGCG therapy in the future, but rather used for the experimental simulation of in vivo proteolytic conditions. Evidence from this study indicates that this methodology not only facilitates sustained EGCG release but also simulates a proteolytic environment akin to physiological conditions, potentially elevating the therapeutic applicability of PG matrices in mitigating oxidative stress-related cellular damage. In addition, this research expands the possibilities for EGCG delivery systems and emphasizes the importance of convenient and cost-effective drug-delivery methods in current and future pharmaceutical research [18,19].

### 4.1. Applicability of the Spectrophotometric Assay for EGCG Levels in the Experimental System

In general, conventional methods for the quantitative determination of EGCG use high- (or ultra-high-) performance liquid chromatography alone or in combination with mass spectrometry [20,21,22,23]. However, although sample types may be limited, a simple and convenient method using a spectrophotometer is available without expensive analytical devices [17]. This study did not require the separation of EGCG from other catechin species. Thus, the authors focused on this method and validated its applicability with minor modifications. In a preliminary study, it was observed that reproducible data can be obtained with sufficient vortexing and incubation following the addition of L-arginine. This amino acid can stabilize proteins against aggregation [24].

### 4.2. Manner of EGCG Release from the PG Matrix

EGCG release was evaluated using the in vitro experimental system described in this study. Based on the experience of the polyP release [15], the authors hypothesized that 20−40% of EGCG would be released within the initial phase. However, EGCG in low levels in the PG matrix was not significantly released in the absence of trypsin during the incubation period. Trypsin was added to PBS to simulate in vivo proteolytic conditions. Because plasma (PPP) is expected to stop the proteolytic action of trypsin [25], as serum is used in the process of passage in cell culture, relatively high concentrations of trypsin were used. As expected, trypsin degraded the PG matrix in a dose- and time-dependent manner and released EGCG. The optimal concentration of trypsin was 0.025% after 8 days of incubation.

These quantitative statistical analyses confirm enhanced EGCG release kinetics in the presence of trypsin across the 8-day incubation period. The stepwise significant increases over time suggest the sustained, controlled release of EGCG from the PG matrix when proteolytic degradation occurs. In the human body, trypsin is not the main factor that breaks down the fibrin matrix, regardless of the status of the protein, be it natured or denatured. Instead, many proteolytic enzymes, such as plasmin and immune reactions, including macrophages and lymphocytes, are thought to be involved in this degradation in vivo. However, as long as EGCG is not damaged or highly oxidized in this process, EGCG released from the PG matrix can protect the surrounding cells from reactive oxygen species (ROS), such as hydroperoxide (O_2_H), superoxide (O^2−^), hydroxyl radical (OH·), and singlet oxygen [26].

If EGCG is fully released from the PG matrix without significant degradation in the case of the low EGCG-containing PG matrix, the concentration of EGCG in the PBS can theoretically be approximately 200 μM. However, EGCG release plateaus within 5–6 days at a concentration of roughly 160 μM. This observed difference in EGCG release kinetics, particularly in the presence of trypsin, may represent the levels of degraded EGCG or alternatively underscore the complexity of the interaction between the PG matrix and EGCG, as well as the influence of proteolytic enzymes on this process. This phenomenon raises intriguing questions about the bioavailability of EGCG when delivered through a PG matrix in a physiological setting, where a myriad of proteolytic activities and factors beyond trypsin, such as plasmin and various cellular activities, play a crucial role in material degradation and drug release [27,28].

The increases in EGCG levels added to the PPP fraction were preliminarily found to be easily degradable in PBS at 37 °C. Thus, simply to evaluate the antioxidant capacity of the released EGCG, the high EGCG-containing PG matrix was prepared by means of prolonged heating. As expected, the optically confirmed, non-enzymatic degradation was minimized, and trypsin degraded the matrix time-dependently. Under these conditions, EGCG was released without trypsin immediately after starting incubation, although trypsin significantly increased EGCG release. Based on the calculation, when EGCG is fully released from this PG–EGCG matrix, the EGCG levels in the PBS would be 833 μM. However, the measured EGCG levels reached approximately 200 μM within 7 days. This substantial difference is probably due to the degradation of released EGCG and/or the bonding of released EGCG to particular plasma components.

As described at the beginning of the above paragraph, the purpose of this modification was not to improve the PG matrix but to test the antioxidant capacity of EGCG released from the PG matrix. In fact, the resulting PG–EGCG matrix was not a gel-like soft matter but rather had a heat-compressed tablet-like appearance. Judging from the SEM findings (Figure 3), the incorporation of EGCG molecules into plasma proteins, mainly fibrin, interferes with the polymerization and crosslinking of fibrin fibers, thereby reducing the integrity of the matrix. Owing to such structural defects, this PG–EGCG matrix can be considered unsuitable for topical application by means of injection or implantation.

### 4.3. Antioxidant Capacity of Released EGCG

Moreover, the chemical stability of EGCG and its interaction with the PG matrix are essential areas of research for optimizing its formulation and enhancing the effectiveness of delivery systems [15]. EGCG is extracted from green tea by boiling water and is consumed orally in order to function in the body. Therefore, EGCG is likely chemically stable. However, EGCG is easily degradable at low concentrations under neutral and alkaline conditions and at high temperatures [9]. This characterization predicted that EGCG released from the PG matrix would degrade and lose its antioxidant activity.

In the case of the PG matrix containing low EGCG levels, owing to the high PG antioxidant capacity, it was difficult to detect the EGCG-dependent antioxidant capacity and thereby evaluate how long the released EGCG maintained its antioxidant action, if any. Thus, to distinguish EGCG’s antioxidant capacity from the PG-dependent capacity, the preparation protocol was modified with prolonged heating time and increased EGCG levels. This modification improved the biggest drawback—that is, the high non-enzymatic degradability of the PG matrix with low EGCG levels—and increased the stability of the resulting PG–EGCG matrix in neutral PBS at 37 °C, in which the antioxidant capacity is anticipated to drop to undetectable levels within several hours. However, it did not interfere with the enzymatic digestion of the PG–EGCG matrix.

Because EGCG was not released in a typical time-dependent manner in the modified PG–EGCG matrix, its antioxidant capacity did not increase with time. More importantly, antioxidant capacity did not decrease immediately. Although further investigation is needed to clarify this mechanism and identify the chemical structure of EGCG, i.e., a bound or free state, these findings suggest that EGCG’s antioxidant capacity could be protected by the degraded PG, probably due to the potent antioxidant capacity of particular plasma components.

### 4.4. Anticoagulants

This study did not optimize the anticoagulants, and thus which anticoagulant is suitable for the preparation of PPP and subsequent PG matrix has not yet been validated. In this study, ACD-A was adopted as an anticoagulant based on the proposal by Marx, a distinguished pioneer of PRP therapy, and the current clinicians’ consensus appeared in the survey of the International Society on Thrombosis and Haemostasis for PRP preparation [29,30]. According to the consensus, for biomedical concerns [31], neither EDTA nor heparin was chosen for PRP preparation. Thus, as introduced elsewhere [32,33,34], when both PRP and PPP are prepared simultaneously and combined before thermal treatment and injection, it is better and more convenient to use the same anticoagulant. In addition to ACD-A, sodium citrate may be an alternative. Both ACD-A and citrate are acidic and may be beneficial for the preservation of EGCG.

However, this study does not support a possible pH shift. Such basic investigations are critical for translating these in vitro findings into viable clinical applications, where the therapeutic potential of EGCG can be fully exploited for regenerative medicine, wound healing, and anti-inflammatory purposes [5,35,36].

### 4.5. Future Perspective

This study sheds light on the delicate balance between the physicochemical properties of the PG matrix and its ability to act as a versatile EGCG carrier. This research underscores the importance of understanding biochemical interactions within the delivery system and the physiological context of its application. These findings suggest that biomaterial-based approaches to therapeutic delivery offer a promising avenue for future research, particularly for the development of new compounds that can precisely control the release of bioactive molecules.

However, this biomaterial has not yet been fully developed as an ideal carrier of EGCG. We believe that some optimizations of the PPP preparation protocol, modifications of the gelation protocol, and refinement of the in vitro experimental system are needed to improve its basic characteristics and to shorten the approach to its clinical application.

Although simple and limited, this study could lead to innovative solutions for the treatment of diseases in which oxidative stress and inflammation play key roles [37,38]. More specifically, the growth factors contained in the PG matrix and EGCG are expected to be released with PG degradation and to function cooperatively at the site of implantation. Thus, the healing properties of PG can be exploited by the antioxidant and anti-inflammatory effects of EGCG. This kind of additive or synergistic action may be clearer in the injured tissues or organs of patients with refractory diseases, such as autoimmune diseases, with successful outcomes achieved by suppressing the exacerbation of inflammation.

Because the degraded PG showed significant antioxidant capacity, one may not appreciate the therapeutic advantages of the PG–EGCG complex. However, the plasma’s total antioxidant capacity varies among individuals [39]. Aging and diet severely influence capacity [40,41,42]. Thus, elderly patients with chronic inflammation and without appropriate dietary intake are thought to be poorly resistant to ROS damage. In addition, it is likely that their plasma antioxidant capacity is sustained at lower levels. Therefore, in patients with a low plasma total antioxidant capacity, the topical application of PE-EGCG complex might be beneficial for suppressing inflammation and potentializing spontaneous tissue regeneration activity.

Nevertheless, advancements in personalized medicine require the integration of autologous biomaterials with pharmacological insights, resulting in safer and more effective therapeutic interventions. Thus, in addition to the basic matters, further preclinical investigations are required to realize its clinical application.

## 5. Conclusions

The thermally prepared PG matrix appeared to function as an efficient and effective carrier for sustained delivery of EGCG. This opens up new possibilities in the fields of regenerative medicine and tissue engineering. Utilizing the biocompatibility and adjustable properties of the PG matrix, this method presents a promising approach for targeted and controlled therapeutic interventions. The ability to regulate the release rate of EGCG, along with the matrix’s innate biodegradability, makes the PG–EGCG complex a significant step forward in drug delivery. Further research is necessary to optimize this delivery platform, expand its application to a wider range of clinical conditions, and eventually move from the laboratory to clinical settings.

## Data Availability

Data are available from the corresponding author upon request.
